# Modeling the first wave of Covid-19 pandemic in the Republic of Cyprus

**DOI:** 10.1038/s41598-021-86606-3

**Published:** 2021-04-01

**Authors:** Sergios Agapiou, Andreas Anastasiou, Anastassia Baxevani, Christos Nicolaides, Georgios Hadjigeorgiou, Tasos Christofides, Elisavet Constantinou, Georgios Nikolopoulos, Konstantinos Fokianos

**Affiliations:** 1 Department of Mathematics and Statistics, University of Cyprus, Nicosia, Cyprus; 2 Department of Business and Public Administration, University of Cyprus, Nicosia, Cyprus; 3 Medical School, University of Cyprus, Nicosia, Cyprus; 4 Cyprus Ministry of Health, Nicosia, Cyprus; 5 Nireas Research Centre, University of Cyprus, Nicosia, Cyprus

**Keywords:** Diseases, Medical research, Mathematics and computing

## Abstract

We present different data analytic methodologies that have been applied in order to understand the evolution of the first wave of the Coronavirus disease 2019 in the Republic of Cyprus and the effect of different intervention measures that have been taken by the government. Change point detection has been used in order to estimate the number and locations of changes in the behaviour of the collected data. Count time series methods have been employed to provide short term projections and a number of various compartmental models have been fitted to the data providing with long term projections on the pandemic’s evolution and allowing for the estimation of the effective reproduction number.

## Introduction

Coronavirus disease 2019 (COVID-19), an infection caused by the novel coronavirus SARS-CoV-2^1^ that first emerged in Wuhan, China^2^ , by the beginning of February 2021, counts more than 105 million cases and has claimed nearly 2,300,000 lives^3^. Despite some advances in therapy^4^ and some vaccines receiving conditional approval^5–7^, there are still many gaps in our understanding of the dynamics of the new pandemic disease, of the effect of the interventions different governments have imposed, and of a number of other epidemiological parameters. Epidemic modeling is a fundamental component of epidemiology, especially with regards to infectious diseases. Following the pioneering work of R. Ross, W. Kermack, and McKendrick in early twentieth century^8^, the discipline has established itself and comprises a major source of information for decision makers. For instance, in the United Kingdom, the Scientific Advisory Group of Emergencies (SAGE) is a major body that collects evidence from multiple sources including inputs from mathematical modeling to advise the British government on its response to the complex COVID-19 situation; for more information see https://www.gov.uk/government/collections/scientific-evidence-supporting-the-government-response-to-coronavirus-covid-19.

In the context of the COVID-19 pandemic, expert opinions can help decision makers comprehend the status of the pandemic by collecting, analyzing, and interpreting relevant data and developing scientifically sound methods and models. An exact model that would describe perfectly the data is usually not feasible and of limited scope; hence scientists usually aim for models that allow a statistical simulation of synthetic data. At the same time, models can also approximate the dynamics of the disease and discover important patterns in the data. In this way, researchers can study various scenarios and understand the likely consequences of government interventions.

The Republic of Cyprus is an island-state, located in the Mediterranean in the southeast of Europe. The first (imported) cases of COVID-19 appeared in early March 2020 with the first wave peaking in late March-early April. The health authorities responded rapidly and rigorously to the COVID-19 pandemic by scaling-up testing, increasing efforts to trace and isolate contacts of cases, and implementing measures such as closures of educational institutions, and travel and movement restrictions. Lacking a scientific unit specialised in epidemics and other infectious health hazards, a diverse group of experts from various disciplines, including epidemiologists, clinicians, statisticians and data scientists was formed with the aim of trying to understand the evolution of the COVID-19 pandemic in Cyprus and of assisting the Cypriot government in informed decision making. This manuscript contains the work of this group, including results from statistical and mathematical models used to understand the epidemiology of the first wave of COVID-19 in Cyprus, which spans from the beginning of March till the end of May 2020. Specifically, we proposed a range of different models that captured different aspects of the COVID-19 pandemic. The analysis consisted of several methods applied to understand the evolution of pandemics in the long and short run. We used change-point detection, count time series methods and compartmental models for short and long term projections, respectively. We estimated the effective reproduction number by using three different methods and obtained consistent results across all the used methods. Results were cross-validated against observed data. Besides providing a comprehensive data analysis, we illustrate the importance of mathematical models to epidemiology.

## Cyprus COVID-19 surveillance system

The Unit for Surveillance and Control of Communicable Diseases (USCCD) of the Ministry of Health operates COVID-19 surveillance. The lab-based surveillance system consisted, during the first pandemic wave, of 19 laboratories (7 public and 12 private) that carried out molecular diagnostic testing for SARS-CoV-2. Sociodemographic, epidemiological, and clinical data of individuals with SARS-CoV-2 infection were routinely collected from laboratories and clinics, and reported to an electronic platform of the USCCD. A confirmed COVID-19 case was a person, symptomatic or asymptomatic, with a respiratory swab (nasopharynx and/or pharynx) positive for SARS-CoV-2 by a real-time reverse-transcription polymerase chain (rRT-PCR) assay. Cases were considered imported if they had travel history from an affected area within 14 days of the disease onset. Locally-acquired cases were individuals who tested positive for SARS-CoV-2 and had the earliest onset date in Cyprus without travel history from affected areas. People with symptomatic COVID-19 were considered recovered after the resolution of symptoms and two negative tests for SARS-CoV-2 at least 24-h apart from each other. For asymptomatic cases, the negative tests to document virus clearance were obtained at least 14 days after the initial positive test. A person with a positive test at 14 days was further isolated for one week and finally released at 21 days after the initial diagnosis without further laboratory tests. Testing approaches in the Republic of Cyprus included: (a) targeted testing of suspect cases and their contacts; of repatriates at the airport and during their 14-day quarantine; of teachers and students when schools re-opened in mid-May; of employees in essential services that continued their operation throughout the first pandemic wave (e.g. customer services, public domain, etc); and of health-care workers in public hospitals, and (b) population screenings following random sampling in the general population of most districts and in two municipalities with increased disease burden. By June 2nd 2020, 120,298 PCR tests had been performed (13,734.2 per 100,000 population). Public health measures were taken in 4 phases: Period 1 (10–14 March, 2020) included closures of educational institutions and cancellation of public gatherings (> 75 persons); Period 2 (15–23 March, 2020) involved closure of entertainment areas (for instance, malls, theatres, etc), allowance of 1 person per 8 square meters in public service areas, and restrictions to international travel (for example, access to the Republic of Cyprus was permitted only for specific persons and after SARS-CoV-2 testing); Period 3 (24–30 March, 2020) included closure of most retail services; and Period 4 (31 March–3 May) included the suspension of incoming flights with few exceptions (for instance, repatriated Cypriot citizens), stay at home order, and night curfew.

## Statistical methods

In this section we introduce the different techniques and models that have been used for the modeling and analysis of the COVID-19 infections in Cyprus.

### Change-point analysis and projections

Change-point detection is an active area of statistical research that has attracted a lot of interest in recent years and plays an essential role in the development of the mathematical sciences. A non-exhaustive list of application areas includes financial econometrics^9^, credit scoring^10^, and bioinformatics^11^. The focus is on the so-called *a posteriori* change-point detection, where the aim is to estimate the number and locations of certain changes in the behaviour of a given data sequence. For a review of methods of inference for single and multiple change-points (especially in the context of time series) under the a-posteriori framework, see^12^. Detecting these change-points enables data segmentation to homogeneous parts thus leading to a more elaborate modeling approach. Advantages of discovering such segments include interpretation and forecasting. Interpretation naturally associates the detected change-points to real-life events or/and political decisions. In this way, improved description of the observed process and the impact of any intervention can be communicated. With regards to forecasting, the role of the final segment is quite important as it allows for a more accurate prediction of future values of the data sequence at hand. Methods developed in this context are based on a given model. For the purpose of this paper, we work with the following signal-plus-noise model

1 $$\begin{aligned} X_t = f_t + \sigma \epsilon _t, \quad t=1,2,\ldots ,T, \end{aligned}$$

where $X_t$ denotes the daily number of COVID-19 cases and $f_t$ is a deterministic signal with structural changes at certain time points. Details about $f_{t}$ are given below. The sequence $\epsilon _t$ consists of independent and identically distributed (iid) data with mean zero and variance equal to one and $\sigma >0$. We denote the number of change-points by *K* and their respective locations by $r_1, r_2, \ldots , r_K$. The locations are unknown and the aim is to estimate them. The daily number of COVID-19 cases in the Republic of Cyprus is investigated by using the following two models for $f_{t}$ of (1):

1. *Continuous, piecewise-linear signals* $f_{t} = \mu _{j,1} + \mu _{j,2}t$, for $t = r_{j-1} + 1, r_{j-1}+2, \ldots , r_{j}$ with the additional constraint of $\mu _{k,1} + \mu _{k,2}r_{k} = \mu _{k+1,1} + \mu _{k+1,2}r_{k}$ for $k=1,2,\ldots ,K$. The change-points, $r_k$, satisfy $f_{r_k-1} + f_{r_k+1}\ne 2f_{r_k}$.
2. *Piecewise-constant signals* $f_t = \mu _j$ for $t = r_{j-1}+1,r_{j-1}+2,\ldots ,r_j$, and $f_{r_j}\ne f_{r_j+1}.$

In this work, we are using the Isolate-Detect (ID) methodology of Anastasiou and Fryzlewicz (2019)^13^ in order to detect changes based on (1) under the settings of continuous piecewise-linear and piecewise-constant signals, as described above; see Online Appendix A1 for a description of the method.

### Count time series methodology

The analysis of count time series data (like daily incidence data we consider in this work) has attracted considerable attention, see Kedem and Fokianos (2002)^14^ for several references and Fokianos (2015)^15^ for a recent review of this research area. In what follows, we take the point of view of generalized linear modeling as advanced by^16^. This framework naturally generalizes the traditional ARMA methodology and includes several complicated data generating processes besides count data such as binary and categorical data. In addition, fitting of such models can be carried out by likelihood methods; therefore testing, diagnostics and all type of likelihood arguments are available to the data analyst.

The logarithmic function is the most popular link function for modeling count data. In fact, this choice corresponds to the canonical link of generalized linear models. Suppose that $\{X_t \}$ denotes a daily incidence time series and assume, that given the past, $X_{t}$ is conditionally Poisson distributed with mean $\lambda _{t}$. Define $\nu _{t} \equiv \log \lambda _{t}$. A log-linear model with feedback for the analysis of count time series^17^ is defined as

2 $$\begin{aligned} \nu _{t} = d+a_{1} \nu _{t-1} + b_{1} \log (X_{t-1}+1). \end{aligned}$$

In general, the parameters $d,a_{1},b_{1}$ can be positive or negative but they need to satisfy certain conditions to obtain stability of the model. The inclusion of the hidden process makes the mean of the process to depend on the long-term past values of the observed data. Further discussion on model (2) can be found in Online Appendix A2 which also includes some discussion about interventions. An intervention is an unusual event that has a temporary or a permanent impact on the observed process. Computational methods for discovering interventions, in the context of (2), under a general mixed Poisson framework have been discussed by^18^. In this work, we will consider additive outliers (AO) defined by

3 $$\begin{aligned} \nu _{t} = d+a_{1} \nu _{t-1} + b_{1} \log (X_{t-1}+1) + \sum _{k=1}^{K}\gamma _{k} I(t= r_{k}) \end{aligned}$$

where the notation follows closely that of the section above and *I*(.) denotes the indicator function. Inclusion of the indicator function shows that at the time point $r_{k}$, the mean process has a temporary shift whose effect is measured by the parameter $\gamma _{k}$ but in the log-scale. Other type of interventions can be included (see Online Appendix A2) whose effect can be permanent and, in this sense, intervention analysis and change-point detection methodologies address similar problems but from a different point of view. Model fitting is based on maximum likelihood estimation and its implementation has been described in detail by Liboschik et al. (2017)^18^.

### Compartmental models

Compartmental models in epidemiology, like the Susceptible-Infectious-Recovered (SIR) and Susceptible-Exposed-Infectious-Recovered (SEIR) models and their modifications, have been used to model infectious diseases since the early 1920’s (see^19,20^ among others). The basic assumptions for these models are the existence of a closed community, i.e. without influx of new susceptibles or mortality due to other causes, with a fixed population, say *N*, and also that the individuals who recover from the illness are immune and do not become susceptible again. In the basic SEIR model, at any point in time *t*, each individual is either susceptible (*S*(*t*)), exposed (*E*(*t*)), infectious (*I*(*t*)) or recovered (*R*(*t*), including death). Typically, the epidemic starts at time $t=0$ with a number of infectious individuals, usually thought of as being externally infected, and the rest of the population being susceptible. People progress between the different compartments and this motion is described usually through a system of ordinary differential equations that can be put in a stochastic framework.

A variety of SEIR modifications and extensions exist in the literature, and a multitude of them emerged recently because of the COVID-19 epidemic. In this work, we consider four such modifications, based on the models proposed in^21^ and^22^ for the analysis of the COVID-19 epidemic in Wuhan and the rest of the Chinese provinces.

#### Compartmental model 1

Initially, we employ the SEIR model based on the metapopulation model of Li et al. (2020)^22^ but simplified to take into account only a single population. The novelty compared to the standard SEIR model, is that this model takes into account the existence of undocumented/asymptomatic infections, which transmit the virus at a potentially reduced rate. The model tracks the evolution of four state variables at each day *t*, representing the number of susceptible, exposed, infected-reported and infected-unreported individuals, $S(t), E(t), I^r(t), I^u(t)$ respectively. The parameters of the model are the transmission rate $\beta$ ($\hbox {days}^{-1}$), the relative transmission rate $\mu$ representing the reduction in transmission for asymptomatic individuals, the average latency/incubation period *Z* (days), the average infectious period *D* (days) and the reporting rate $\alpha$ representing the proportion of infected individuals which are reported. For a graphic description of the model see Figure A4 in the Online Appendix. The time evolution of the system is defined by the following set of differential equations (recall *N* denotes the population size):

4 $$\begin{aligned} \begin{aligned} \frac{d S(t)}{dt}&=-\frac{\beta S(t) I^r(t)}{N}-\frac{\mu \beta S(t) I^u(t)}{N}, \\ \frac{dE(t)}{dt}&=\frac{\beta S(t) I^r(t)}{N}+\frac{\mu \beta S(t) I^u(t)}{N} - \frac{E(t)}{Z}, \\ \frac{dI^r(t)}{dt}&=\alpha \frac{E(t)}{Z}-\frac{I^r(t)}{D},\\ \frac{dI^u(t)}{dt}&=(1-\alpha )\frac{E(t)}{Z}-\frac{I^u(t)}{D}. \end{aligned} \end{aligned}$$

Following Li et al. (2020)^22^, we use a stochastic version of this model with a delay mechanism. Each term, say *U*, on the right hand side of (4) is replaced by a Poisson random variable with mean *U*. At each day, we use the 4th order Runge–Kutta numerical scheme to integrate the resulting equations and obtain the values of the four state variables on the next day. For each new reported infection, we draw a Gamma random variable with mean $\tau _d$ days, to determine when this infection will be recorded. For the main analysis we use $\tau _d$=6 days, as the average reporting delay between the onset of symptoms and the recording of an infection; see also^22^. Note that the results are robust with respect to the value of the reporting delay. The final output of this model is the number of recorded infections on each day *t*, $y=y(t)$.

#### Compartmental model 2

We also use the metapopulation model of Li et al. (2020)^22^. Such metapopulation models have been successfully applied to predict epidemic spreading through human mobility networks^23,24^, by modeling the transmission dynamics in a set of populations, indexed by *i*, connected through mobility patterns, say $M_{ij}$. This is implemented by incorporating information on human movement between the five main districts of Cyprus: Nicosia, Limassol, Larnaca, Paphos and Ammochostos. In this case, $i=1,2,3,4,5$ and $M_{ij}$ denotes the daily number of people traveling from district *i* to district *j*, $i \ne j$. Such information is based on the 2011 census data obtained from the Cyprus Statistical Service. The time evolution of the four compartmental states in each district *i* is defined by the following set of differential equations:

5 $$\begin{aligned} \begin{aligned} \frac{d S_i(t)}{dt}&=-\frac{\beta S_i(t) I_i^r(t)}{N_i}-\frac{\mu \beta S_i(t) I_i^u(t)}{N_i} +\theta \sum _j{\frac{M_{ij}S_j(t)}{N_j-I_j^r(t)}} - \theta \sum _j{\frac{M_{ji}S_i(t)}{N_i-I_i^r(t)}}, \\ \frac{dE_i(t)}{dt}&=\frac{\beta S_i(t) I_i^r(t)}{N_i}+\frac{\mu \beta S_i(t) I_i^u(t)}{N_i} - \frac{E_i(t)}{Z} +\theta \sum _j{\frac{M_{ij}E_j(t)}{N_j-I_j^r(t)}} - \theta \sum _j{\frac{M_{ji}E_i(t)}{N_i-I_i^r(t)}}, \\ \frac{dI_i^r(t)}{dt}&=\alpha \frac{E_i(t)}{Z}-\frac{I_i^r(t)}{D}, \\ \frac{dI_i^u(t)}{dt}&=(1-\alpha )\frac{E_i(t)}{Z}-\frac{I_i^u(t)}{D} +\theta \sum _j{\frac{M_{ij}E_j(t)}{N_j-I_j^u(t)}} - \theta \sum _j{\frac{M_{ji}E_i(t)}{N_i-I_i^u(t)}}, \end{aligned} \end{aligned}$$

where the notation follows the notation given in Compartmental Model 1 Section. In addition to the four state variables, this model also updates at each time step the population of each area *i*, say $N_i$, by $N_i = N_i + \theta \sum _j{M_{ij}} - \theta \sum _j{M_{ji}}$, where the multiplicative factor $\theta$ is assumed to be greater than 1 to reflect under-reporting of human movement. Like Model (4), Model (5) is integrated stochastically using a 4th order Runge-Kutta (RK4) scheme. Specifically, for each step of the RK4 scheme, each unique term on the right-hand side of the four equations is determined using a random sample from a Poisson distribution. The equations describing the evolution of the population in each district *i*, are solved deterministically, $i=1,\ldots ,5.$ For more, see the supplement of Li et al. (2020)^22^.

#### Compartmental model 3

Further, we consider the meta-population model of Peng et al. (2020)^21^. This is a generalisation of the classical SEIR model, consisting of seven states: (*S*(*t*), *P*(*t*), *E*(*t*), *I*(*t*), *Q*(*t*), *R*(*t*), *D*(*t*)). At time *t*, the *susceptible* cases *S*(*t*) will become with a rate $\zeta$ *insusceptible* *P*(*t*) or with a rate $\beta$ *exposed* *E*(*t*), that is infected but not yet infectious, i.e. in a latent state. Some of the exposed cases will eventually become *infected* with a rate $\gamma$. Infected means they have the capacity of infecting but are not *quarantined* yet. The introduction of the new quarantined state, *Q*(*t*), in the classical SEIR model, formed by the infected cases with a constant rate $\delta$, allows to consider the effect of preventive measures. Finally, the quarantined cases, are now split to *cured* cases, *R*(*t*), with rate $\lambda (t)$ and to *closed*, *D*(*t*), with mortality rate $\kappa (t)$. The model’s parameters are the transmission rate $\beta$, the protection rate $\zeta$, the average latent time $\gamma ^{-1}$ (days), the average quarantine time $\delta ^{-1}$ (days) as well as the time dependent cure rate $\lambda (t)$ and mortality rate $\kappa (t)$. The relations are characterized by the following system of differential equations:

6 $$\begin{aligned} \begin{aligned} \frac{d S(t)}{dt}&=-\frac{\beta S(t) I(t)}{N}-\zeta S(t),&\frac{dE(t)}{dt}&=\frac{\beta S(t) I(t)}{N}-\gamma E(t),\\ \frac{dI(t)}{dt}&=\gamma E(t)-\delta I(t),&\frac{dQ(t)}{dt}&= \delta I(t)-\lambda (t) Q(t)-\kappa (t) Q(t),\\ \frac{dR(t)}{dt}&= \lambda (t) Q(t),&\frac{dD(t)}{dt}&= \kappa (t) Q(t),\\ \ \frac{dP(t)}{dt}&=\zeta S(t). \end{aligned} \end{aligned}$$

The total population size is assumed to be constant and equal to $N=S(t)+P(t)+E(t)+I(t)+Q(t)+R(t)+D(t)$. According to the official reports, the number of quarantined cases, recovered and deaths, due to COVID-19, are available. However, the recovered and death cases are directly related to the number of quarantined cases, which plays an important role in the analysis, especially since the numbers of exposed (*E*) and infectious (*I*) cases are very hard to determine. The latter two are therefore treated as hidden variables. This implies that we need to estimate the four parameters $\zeta ,\beta , \gamma ^{-1}, \delta ^{-1}$ and both the time dependent cure rate $\lambda (t)$ and mortality rate $\kappa (t)$. Notice here that while the rest of the parameters are considered fixed during the pandemic, we allow the cure and mortality rate to vary with time. We expect that the former will increase with time, given that social distancing measures have been put in place, while the latter will decrease. Finally, this is an optimization problem, and the methodology we have followed in order to address it can be found in Online Appendix A3.

#### Compartmental model 4

The last model we consider is a modified version of a solution created by Bettencourt and Ribeiro (2008)^25^ to estimate real-time effective reproduction number $R_{t}$ (To avoid confusion, the notation $R_t$ denotes the effective reproduction number but *R*(*t*) denotes the number of recovered cases in a given population) using a Bayesian approach on a simple Susceptible - Infected (SI) compartmental model:

7 $$\begin{aligned} \begin{aligned} \frac{d S(t)}{dt}&=-\frac{\beta S(t) I(t)}{N},\\ \frac{dI(t)}{dt}&=\frac{\beta S(t) I(t)}{N}-\frac{I(t)}{D}.\\ \end{aligned} \end{aligned}$$

We use the Bayes rule to update the beliefs about the true value of $R_t$ based on our predictions and on how many new cases have been reported each day. Having seen *k* new cases on day *t*, the posterior distribution of $R_t$ is proportional to (denoted by $\propto$) the prior beliefs of the value of $P(R_t)$ times the likelihood of $R_t$ given that we have recorded *k* new cases, i.e., $P(R_t | k) \propto P(R_t) \times L(R_t | k)$. To make this iterative every day that passes by, we use last day’s posterior $P(R_{t-1} | k_{t-1})$ to be today’s prior $P(R_t)$. Therefore in general $P(R_t | k) \propto \prod _{t=0}^{T}{L(R_t | k_t)}$. However, in the above model the posterior is influenced equally by all previous days. Thus, we propose a modification suggested in^26^ that shortens the memory and incorporates only the last *m* days of the likelihood function, $P(R_t | k) \propto \prod _{t=m}^{T}{L(R_t | k_t)}$. The likelihood function is modelled with a Poisson distribution.

### Estimation of the effective reproduction number $R_t$

Recall the compartmental models discussed in the Compartmental Model 1 and 2 sub-sections. Then the effective reproduction number is given by

8 $$\begin{aligned} R_t=\alpha \beta D + (1-\alpha )\mu \beta D, \end{aligned}$$

see the supplement of Li et al.^22^. We estimate $R_t$ in (8) during consecutive time periods (either week-long or fortnight-long) for which its value is considered to be constant. To achieve this we estimate the parameters of each model, also assumed to be constant for each time period, using the daily number of diagnoses in the Republic of Cyprus (In order to maintain consistent notation throughout this article, we use the notation $R_t$, even though in this section the effective reproduction number is considered constant for each time period).  To estimate the parameters we employ Bayesian statistics, that is, we postulate prior distributions on the parameters and incorporate the data and the model (through the likelihood) to obtain the posterior distributions on the parameters. The posterior distributions capture our updated beliefs about the parameters after combining the prior with the observed data; see for example^27^.

For the model defined by (4), we consider the whole area of Cyprus as a single uniform population. For this case, the observations are not sufficiently informative to identify all five parameters of the model. A solution would be to enforce identifiability by postulating strongly informative prior distributions on the parameters. Instead, we choose to make the assumption that the parameters *Z*, *D* and $\mu$ have globally constant values, fixed over time. In particular we set $D=3.5$ and $\mu =0.5$ as estimated in^22^ and $Z=5.1$ which appears to be the globally accepted mean incubation period. We thus only need to infer the reporting rate $\alpha$ and the transmission rate $\beta$, which vary both temporally and between different countries because of the amount of testing and the degree of adherence to the social distancing policies. On the other hand, the model defined by (5) is sufficiently informative to infer all six model parameters. All computational methods, prior modelling and assumptions in relation to both compartmental models discussed in Compartmental Model 1 and 2 sub-sections are given in Online Appendix A4. In addition to the above methods we further consider the method of Corie et al. (2013)^28^ as a benchmark to compare all methodologies for estimating the effective reproduction number.

## Results

### Descriptive surveillance statistics

**Figure 1:**
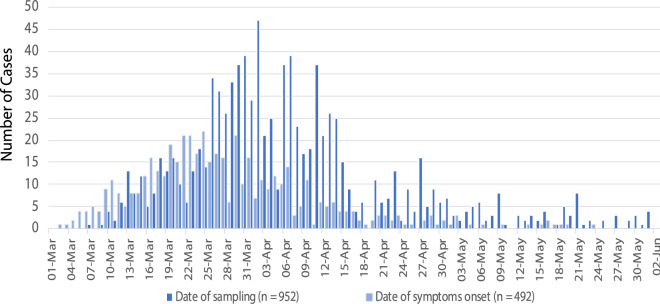
The epidemic curve by date of sampling and date of symptom onset during the observation period.

By the end of May 2020, 952 cases of COVID-19 were diagnosed in the Republic of Cyprus. Of these, 50.2% were male ($n = 478$) and the median age was 45 years (IQR: 31–59 years). The setting of potential exposure was available for 807 cases (84.8%). Of these, 17.4% ($n = 140$) had history of travel or residence abroad during a 14-day period before the onset of symptoms. Locally acquired infections were 667 (82.7%) with 8.6% ($n = 57$) related to a health-care facility in one geographical setting (cluster A) and 12.4% ($n = 83$) clustered in another setting (cluster B). The epidemic curve by date of sampling and date of symptom onset is shown in Fig. 1. The number of cases started to decline in April reaching very low levels in late May.

### Long-term impact of the COVID-19 epidemic on Cyprus

In this section, we investigate the long-term impact of COVID-19 to Cyprus. Towards this, we give long-term projections for the daily incidence and death rates. We fit system (6) to COVID-19 data that were collected during the period from the 1st of March 2020 till the 31st of May 2020, in Cyprus. We treat all the reported cases without making the distinction between *local* and *imported*. The model parameters are estimated using the methodology described in Online Appendix A3. When the model is fitted to data, it can be used to forecast the epidemic. In order to study the evolution of the model as new data are added and the quality of the respective forecasts, we have fitted model (6) in four datasets constructed from the original one using different time periods. Specifically, the four datasets were formed using the daily reported numbers of diagnoses from the beginning of the observation period until and including the 2/4/2020, 17/4/2020, 15/5/2020 and 24/5/202 respectively. The dates were chosen according to the change points detected using the methodology described in Change-point Analysis and Projection Section.

**Figure 2:**
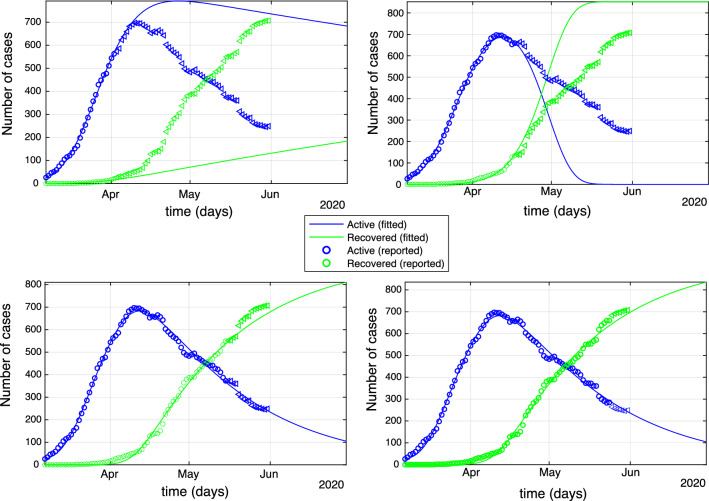
Predictions obtained after fitting (6) for the number of active cases (green) and recovered cases (blue) until the end of the observation period. Parameter estimation was performed by subsampling the data until (top left) 2/4/2020, (top right), 15/4/2020, (bottom left) 15/05/2020 (bottom right) 24/5/2020.The relative error equals $0.4\%, 0.2\%, 0.3\%, 0.3\%$ for the recovered and $18\%, 5.8\%, 0.16\%, 0.1\%$ for the active cases.

The fitted model in each case was used in order to predict the pandemic’s evolution until the 30/6/2020. In Fig. 2, we show the number of predicted exposed plus infectious cases (green solid lines) and the number of predicted recovered cases (blue solid lines) for the duration of the prediction period, and compare them to the observed cases which are indicated by circles and triangles. We use circles for data that have been used in the prediction and triangles for the observed data that are used for validation. Visual inspection shows that after a period of about two months during which the model overestimates the number of active cases and underestimates the number of recovered, see Fig. 2 (top), model (6) was able to capture accurately the evolution of the pandemic, Fig. 2 (bottom).

The performance of the predictions can also be evaluated by means of the relative error (RE) which are computed using $RE=\sqrt{\frac{\sum _{t}(y_t-x_t)^2}{\sum _{t} x_t^2 }}$, where $x_t$ denotes the datum for day *t* and $y_t$ the model prediction for the same day. The RE for the recovered cases equals $0.4\%, 0.2\%, 0.3\%$ and $0.3\%$ for the four time periods respectively with the corresponding RE for the active cases being high in the beginning $18\%, 5.8\%$, but then dropping considerably $0.16\%$ and $0.1\%$, reflecting the fact the model caught up with the evolution of the pandemic. Overall, system (6) gives adequate predictions especially when data from longer time periods are used.

**Figure 3:**
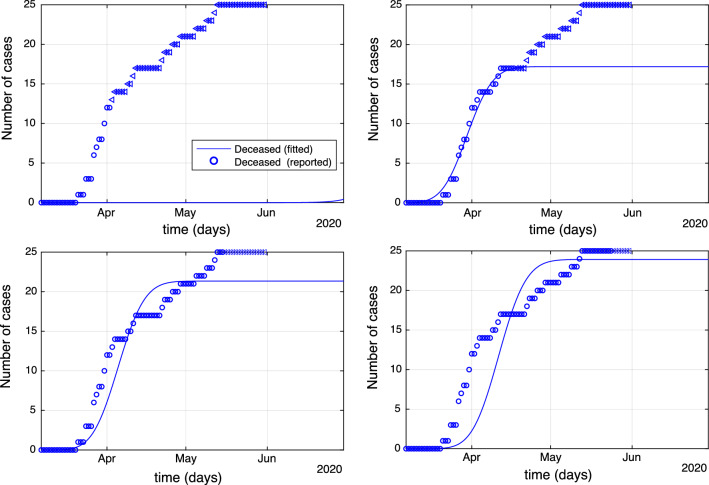
Predictions obtained after fitting (6) for the number of deaths until the end of the observation period. Parameter estimation was performed by subsampling the data until 2/4/2020 (top left), 15/4/2020 (top right), 15/05/2020 (bottom left), 24/5/2020 (bottom right). Corresponding RE’s equal $2\%, 4\%$ and $8\%$ clockwise from top right to bottom right.

Figure 3 shows the number of deaths and their respective predictions using subsets of data as described above. In the duration of the first data set, there were no deaths registered and therefore the prediction was identically zero, giving also an RE equal to $100\%$ see Fig. 3 (top left). As more deaths are registered the model’s ability to predict the correct number of deaths is improving, see Fig. 3.

The recovery rate ($\lambda (t)$) is modelled as

9 $$\begin{aligned} \lambda (t)=\frac{\lambda _1}{1+\exp (-\lambda _2(t-\lambda _3))}, \quad \lambda _i\ge 0, \quad i=1,2,3. \end{aligned}$$

**Figure 4:**
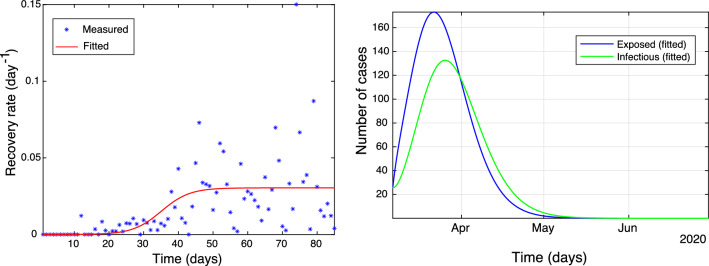
(Left plot ) The recovery rate ($\hbox {days}^{-1}$) (blue stars) and the corresponding fitted function (9) (red solid line). (Right plot) Exposed *E*(*t*) and infectious *I*(*t*) cases.

The idea is that the recovery rate, as time increases, should converge towards a constant. In Fig. 4 (left), the fitted recovery rate (solid line) is plotted against the observed number of recovered cases (stars).

Finally, Model 3 can be used to estimate the unobserved number of exposed, *E*(*t*), and infectious, *I*(*t*), cases during the development of the pandemic. The maximum number of exposed cases occurs on the 21st of March 2020 and is estimated to be 173 cases, Fig. 4 (right, blue line), with the maximum of infectious individuals (136) being attained on the 26th of March 2020. We can observe a delay in the transition of exposed to infectious in the order of 5 days, which suggests a 5 day latent time of COVID-19.

### Change-point analysis

**Figure 5:**
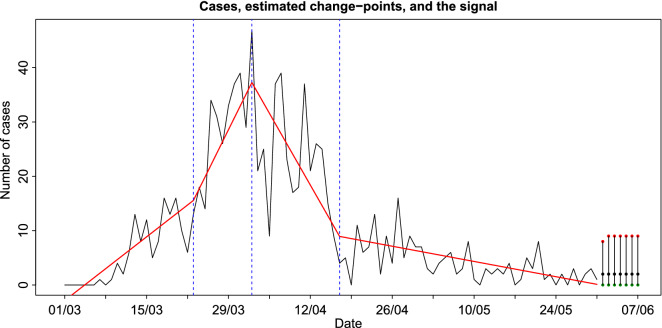
The real data (black coloured line) and the estimated piecewise-linear fit (red coloured line) for the daily incidence rate. The change-point locations are given with dotted, blue vertical lines. At the right-end of the plot point estimators (black dots) and $95\%$ prediction intervals for the number of daily cases for the next week are reported.

We first consider the change-point detection method of the Statistical Methods section for the case of piecewise-linear signal plus noise model. Figure 5 illustrates the results obtained by this analysis on daily incidence data.

This method has detected three important changes. The first change occurs on the 23rd of March, while the last two changes are estimated on the 2nd and 17th of April. The first change-point, on the 23rd of March, indicates a significant increase in the number of cases, and it is believed to be related to the development of the clusters A and B as mentioned in the Descriptive surveillance statistics section. The second change-point shows that the upward trend vanishes and a negative slope takes its place leading to a vast decrease in the number of cases. There is a connection of this change point with the Government’s lockdown decrees on the 24th and 31st of March; it shows the almost immediate impact that such decisions can have in fighting the pandemic. The third change-point indicates stabilisation in the number of the detected cases over the last period. The median for the number of cases in the last segment is equal to 3. Predictions (together with 95% CI.) for the week ahead are also shown in Fig. 5. Note that there will be no more than 8 cases on the 1st of June and no more than 9 cases per day in the period from the 2nd of the month until the 7th. The point estimator is found to be equal to 2 cases per day. The corresponding analysis for the piecewise-constant signal plus noise model is given in Online Appendix A5. Both models describe adequately the daily number of cases and (a) they provide a justification of the positive impact of the Government’s measures in fighting COVID-19, (b) they give a clear view of how contagious the virus is, especially in cases when hubs are developed in the society, and (c) the last homogeneous discovered segment provides better understanding of the current state of the virus in terms of its transmittal.

### Count time series analysis

We first fit model (2) without interventions to daily incidence data, considering the time period between the 4th of March and the 31st of May. Maximum likelihood estimation shows that the fitted model is given by

$$\begin{aligned} {\hat{\nu }}_{t} = -0.003_{(.009)} + 0.547_{(0.447)} {\hat{\nu }}_{t-1}+ 0.451_{(.050)} \log (1+Y_{t-1}). \end{aligned}$$

Corresponding standard errors of the regression coefficients are given underneath in parentheses. Note that the sum of the coefficients $0.547+0.451 \sim 1$ which shows evidence of non-stationarity observed in the data. Exploring further the data by investigating existence of interventions we find two additive outliers on 13th and 26th of March (*p*-value after adjusting for all type of interventions is negligible). The resulting model is given by

$$\begin{aligned} {\hat{\nu }}_{t} =-0.007_{(.007)} + 0.779_{(0.046)} {\hat{\nu }}_{t-1} +0.211_{(.046)} \log (1+Y_{t-1})+1.643_{(0.137)} I(t=10) + 1.102_{(0.288)} I(t=23). \end{aligned}$$

**Figure 6:**
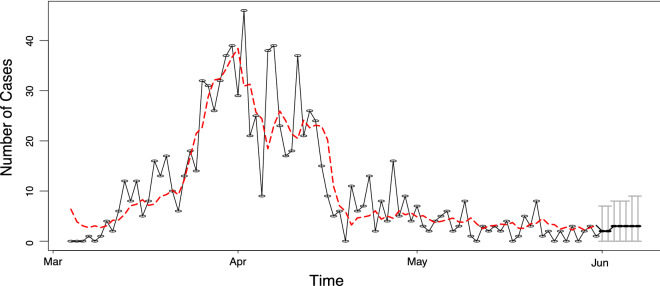
Daily number of COVID-19 cases in Cyprus from 01/03/2020 to 31/05/2020 (black line) and the fitted model with interventions (red line). The grey bars at the end of the plot show 95% simultaneous prediction intervals.

Note again that the sum $0.779+211 \sim 1$ which shows that the non-stationarity persists even after including additive outliers (in the log-scale). Furthermore, the positive sign of both interventions shows the sudden explosion of the daily number of people infected. The corresponding Bayesian information criterion (BIC) values obtained after fitting this model is equal to 576.643 which improves the BIC of the model without intervention which was equal to 615.766. Figure 6 shows the fit of the model to the data and gives 95% prediction intervals for the week ahead.

Comparing both change-point analysis (see Fig. 5) and the result obtained by using the above intervention analysis, we observe that both approaches give similar prediction intervals that include future observed incidence data. Indeed, the observed data for the week ahead (01/06/2020-07/06/2020) were 4,6,1,0,5,5 and 1 cases.

### Results for the effective reproduction number

Recall the effective reproduction number $R_t$ defined by (8). We perform Bayesian analysis using (4) (see Section “Estimation of the effective Reproduction Number $R_t$”) separately on two sets of data: the data concerning all COVID-19 diagnoses in Cyprus and the data concerning diagnoses from local transmission only; for details regarding prior modelling and computation see Online Appendix A4. We examine six consecutive fortnight periods, for each of which we draw 10,000 steps of the used Markov chain Monte Carlo algorithm (independence sampler), discard the first 2000 steps as burn-in and use the remaining ones to approximate the posterior distributions of $\alpha , \beta$ and hence $R_t$.

**Figure 7:**
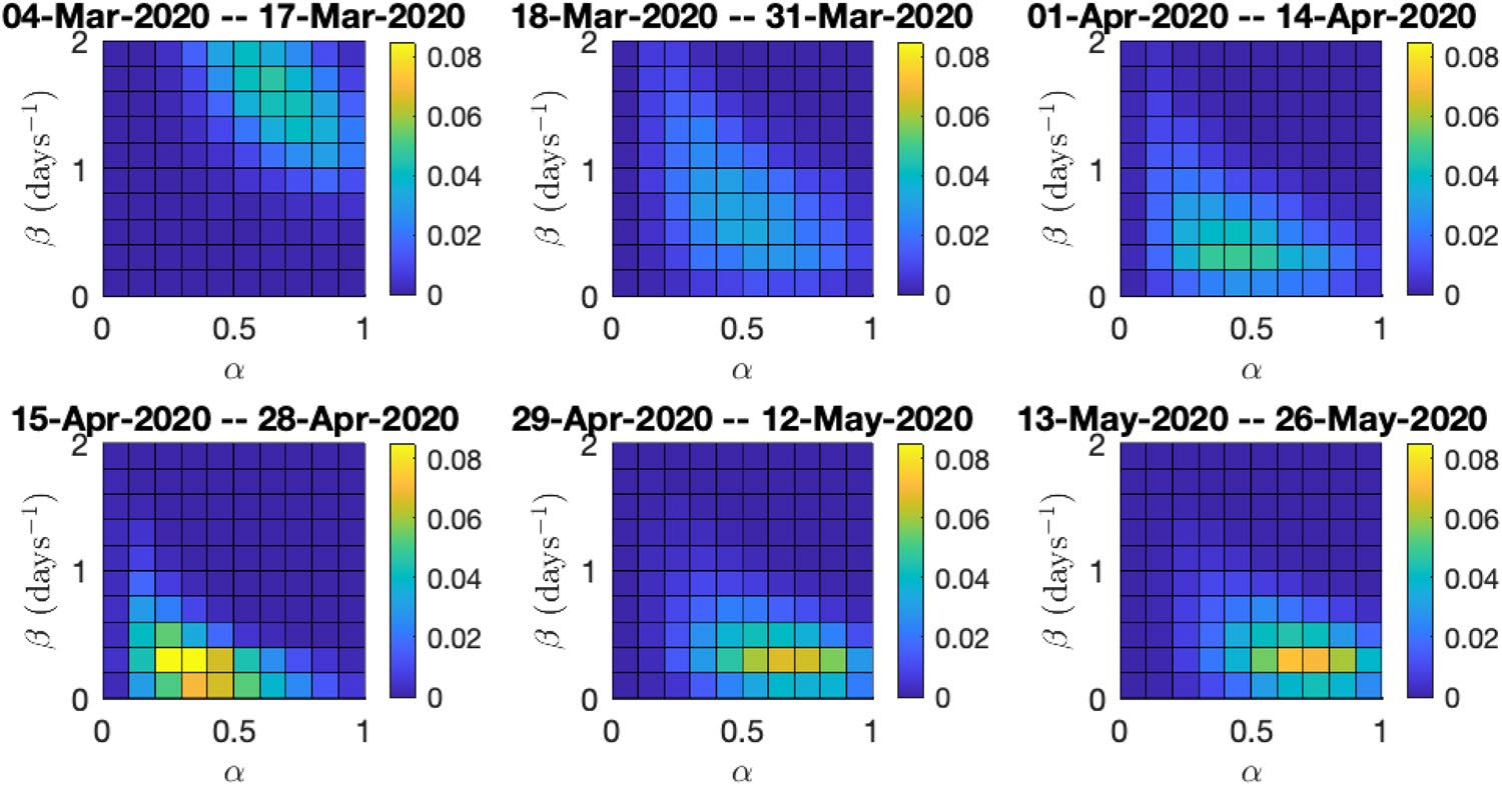
Joint posterior distributions of the reporting rate $\alpha$ and the transmission rate $\beta$ in Model 1, for the six fortnight periods starting from 04/03/2020 until 26/05/2020. Analysis using the full data.

For the data concerning all diagnoses, the first recorded incident was on 07/03/2020, hence, as detailed in Online Appendix A4, we initialize our analysis of the outbreak 3 days earlier, on 04/03/2020. Figure 7 shows the estimated joint posterior distributions of the reporting rate $\alpha$ and the transmission rate $\beta$, for the six fortnight periods. In the first period the posterior probability is distributed in a wide range of values, while in the following periods it concentrates on a narrower range of values. In particular, in the first period, $\beta$ takes with high posterior probability values between 1 and 2, while $\alpha$ concentrates between 0.5 and 1. This can be attributed on both the data and the postulated priors on $\alpha$ and $\beta$. Early in the outbreak, there was a high degree of social interactions, and only a small fraction of the public took protective measures, hence an infected individual could transmit the virus to many people before getting detected and put into isolation; this explains the high values of $\beta$. Furthermore, there were only a few recorded diagnoses, the virus had not yet spread in the community and due to the effective contact tracing procedures, the reporting rate was high; this can explain the high values of $\alpha$ (even though the prior on $\alpha$ at this stage penalizes values far from 0.5). In the next two periods, the introduction of lockdown measures by the government and the adherence of the majority of the public to the advised protective measures, results to lower values of $\beta$. At the same time, the virus has penetrated certain local communities (cf. hubs A and B in the Descriptive surveillance statistics subsection), and as a result we have lower values of the reporting rate $\alpha$, initially around 0.5 and in the third and fourth periods between 0.2 and 0.5 (even though the prior on $\alpha$ at this stage puts higher penalty on small values). In the fourth period, there is a high concentration of the posterior distribution on even lower values of $\beta$. This is the effect of the continued strict lockdown imposed. Finally in the final two periods, the values of $\beta$ remain low, with a slight increase compared to the fourth period, which can be attributed to the relaxation of measures by the government on 04/05/2020. The values of the reporting rate $\alpha$ significantly increase in the last two periods, which can be attributed to the very high number of both targeted and random testing performed.

**Figure 8:**
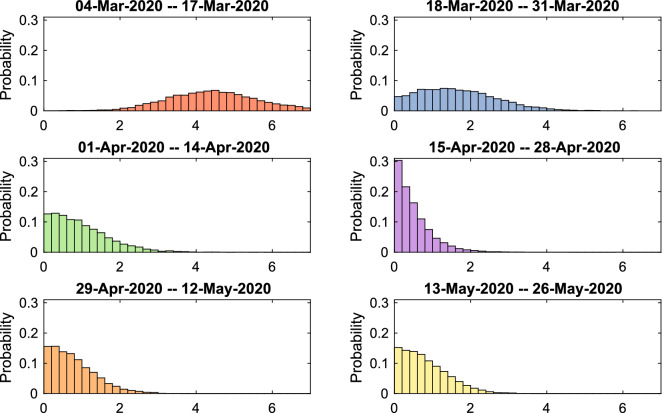
Posterior distributions of the effective reproductive number in Model 1, for the six fortnight periods starting from 04/03/2020 until 26/05/2020. Analysis using the full data.

**Figure 9:**
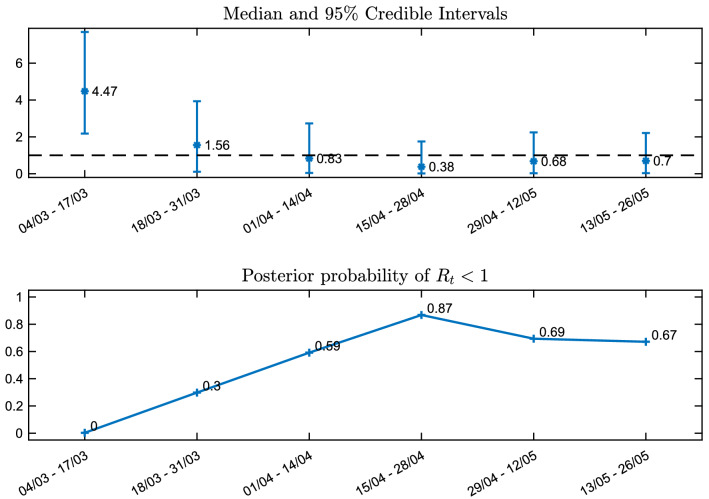
Top: median and 95$\%$ credible intervals for the posterior distributions of the effective reproduction number in Model 1, for the six fortnight periods starting from 04/03/2020 until 26/05/2020. Bottom: posterior probabilities of the event $R_t<1$. Analysis using the full data.

The considerations in the last paragraph, combined with Eq. (8), explain the results on the effective reproduction number $R_t$ in Figs. 8 and 9. In Fig. 8 and the top part of Fig. 9, we see that the posterior distribution of $R_t$, is spread in a wide range of high values between 3–6, with a median of 4.47, while in the next three periods with the introduction of the progressively stricter measures the posterior increasingly concentrates on lower values. In particular in the fourth period, the posterior median is 0.38. In the last two periods, with the relaxation of the lockdown, there is a small increase in the values of $R_t$, however its posterior distribution still mostly concentrates under 1, with medians around 0.7. The bottom part of Fig. 9, shows the posterior probabilities of the event $R_t<1$, which steadily increase following the progressively stricter measures imposed, from 0 in the first period to 0.87 in the fourth period, before slightly dropping to around 0.69 and 0.67 with the relaxation of measures in the last two periods, respectively.

For the data concerning only locally transmitted diagnoses, the first recorded incident was on 10/03/2020, hence we initialize our analysis of the outbreak three days earlier, on 07/03/2020. The results of the analysis using data concerning only local transmission diagnoses, are similar to the ones of the analysis using the full data; see Figures A5, A6 and A7 in the Online Appendix.

Next, we consider the estimation model described in^22^ where Cyprus is divided in five subpopulations (Nicosia, Limassol, Larnaca, Paphos, Ammochostos) and the mobility patterns between them are taken into account (as described in Metapopulation compartmental model 2). The effective reproduction number is given by (8). The compartmental model 2 structure was integrated stochastically using a 4th order Runge-Kutta (RK4) scheme. We use uniform prior distributions on the parameters of the model, with ranges similar to Li et al (2020)^22^ as follows: relative trasmissibility $0.2\le \mu \le 1$, movement factor $1\le \theta \le 1.75$; latency period $3.5\le Z\le 5.5$; infectious period $3\le D \le 4$. For the infection rate we choose $0.1\le \beta \le 1.5$ before the lockdown and $0\le \beta \le 0.8$ after the lockdown and for the reporting rate we choose $0.3\le \alpha \le 1$. Note that the Ensemble Adjustment Kalman Filter (EAKF, described in Online Appendix A4) is not constrained by the initial priors and can migrate outside these ranges to obtain system solutions. For the initialization purposes we assume that all five districts are potential origins with an undocumented infected and exposed population drawn from a uniform distribution [0, 5] a week before the first documented case. Initial condition does not affect the outcome of the inference. Transmission model 2 does not explicitly represent the process of infection confirmation. Thus, we mapped simulated documented infections to confirmed cases using a separate observational delay model. In this delay model, we account for the time interval between a person transitioning from latent to contagious and observational confirmation of that individual infection through a delay of $T_d$. We assume that $T_d$ follows a Gamma distribution $G(a,\tau _d/a)$ where $\tau _d=6$ days and $a=1.85$ as derived by Li et al. (2020)^22^ using data from China. Inference is robust with respect to the choice of $\tau _d$.

**Figure 10:**
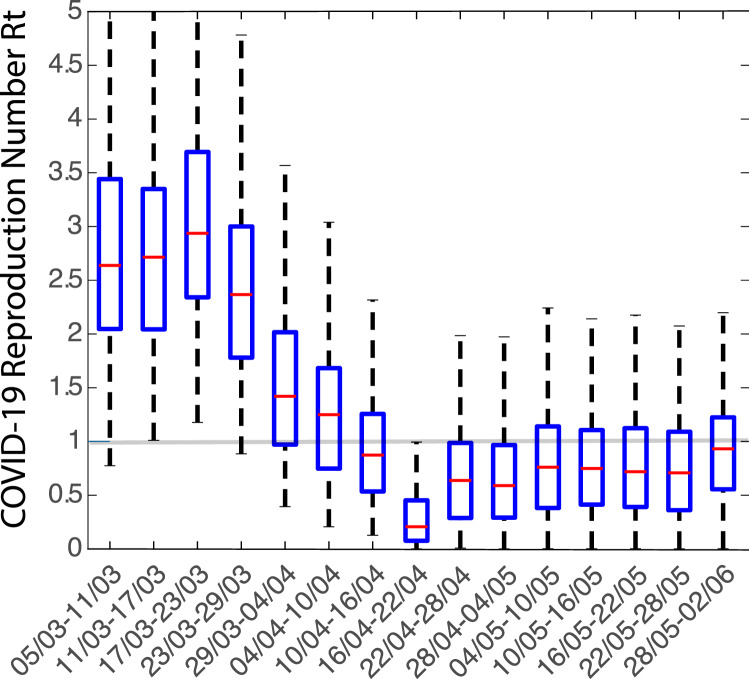
Median value (red) and 95$\%$ credible intervals (box) for the posterior distributions of the effective reproduction number in metapopulation compartmental model 2, for the period between 05/03/2020 and 02/06/2020.

For the inference we use diagnoses from local transmission in Cyprus as were reported by the Ministry of Health. In Fig. 10 we plot the time evolution of the weekly effective reproduction number $R_t$. While at the beginning of the outbreak the effective reproduction number was close to 2.5, after the lockdown measures, it dropped below 1 and stayed consistently there until the end of May 2020.

**Figure 11:**
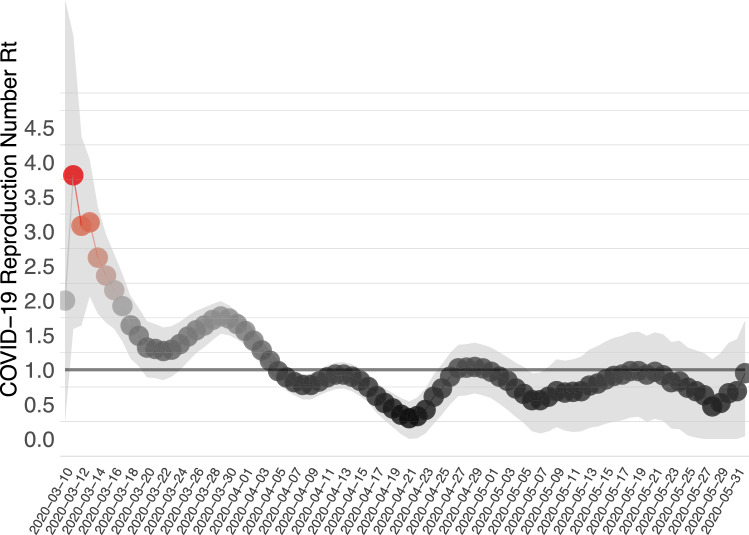
Median value (red) and 95$\%$ credible intervals for the posterior distributions of the effective reproduction number in compartmental model 3, for the period between 05/03/2020 and 01/06/2020.

We then use the methodology proposed by^25^ and recently modified by^26^ as described in detail in Compartmental Model 4 sub-section. For that method we also use the diagnoses from local transmission in Cyprus as were reported by the Ministry of Health. Figure 11 shows the daily median value as well as the 95% credible intervals for the effective reproductive number using that method.

**Figure 12:**
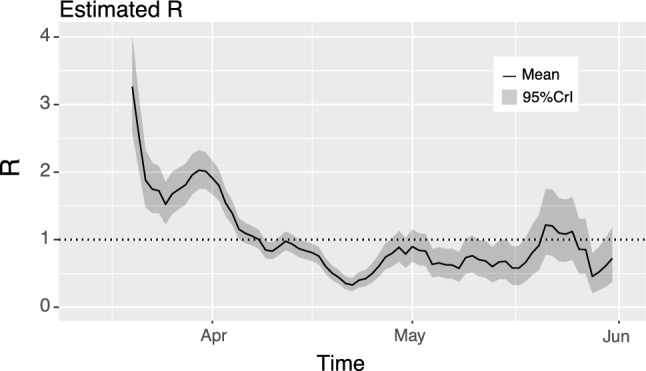
Estimation of $R_{t}$ based on the methodology of^28^ together with 95% confidence (green curve) intervals. The plot is based on weekly data and on choosing the serial interval distribution to be Gamma with mean 6.48 days and standard deviation of 3.83; see^29^ for more.

These results should be compared with the methodology of Corie et al. (2013)^28^—see Fig. 12 which shows weekly estimates of $R_{t}$ based on weekly time intervals. At the end of May, COVID-19 was well contained in Cyprus especially even though the disease initiated with a high value of $R_{t}$. The government lockdown helped reduce the reproduction number, as the data shows. Comparing the results obtained by all methods presented in this Section, we see no gross discrepancies on concluding that $R_{t} < 1$ by the end of May, with high probability.

## Discussion

The work presented in this report is the result of the efforts of the authors to give guidance to the Cypriot government for controlling the COVID-19 infection outbreak. Different models and methods have been applied to the data collected by the Unit for Surveillance and Control of Communicable Diseases of the Cypriot Ministry of Health.

Change point detection has been used in order to estimate the number and locations of changes in the behaviour of data. Three important changes have been detected. The first one indicated a significant increase in the number of cases, that we suspect is related to the formation of two clusters of COVID-19 infection cases, while the other two indicate a drop and stabilisation respectively in the numbers of cases that are probably linked to the effectiveness of the Government’s lockdown decrees. A log-linear model with feedback and additive outliers was utilised to examine the existence of interventions resulting in two cases. Both methods were subsequently used to provide with prediction intervals on the number of future numbers of diagnoses with comparable results.

Modifications of the SEIR model have been employed in order to study the long-term impact of the COVID-19 to Cyprus. As expected, the fitting improved once enough data were available, with a very good agreement to the observations. Using the fitted Model 3 it was possible to additionally estimate the number of unobserved exposed and infectious cases during the pandemic, with the maximum number of infectious individuals being reached on the 26th of March. Moreover the SEIR based Models 1 and 2 facilitated the estimation and prediction of the effective reproduction number. The estimation was performed in a Bayesian framework during consecutive time periods for which the effective reproduction number was considered constant. We have treated two separate cases: in the first, there was no distinction between the patients while in the second we have considered only those patients resulting from local transmission. Finally, we have estimated the effective reproduction number for a partition of the population according to the five geographic areas of Cyprus.

## Methods

All methodology is described in detail in "Results" section.

## Supplementary Information

Supplementary Information.

## Data Availability

Data and code are available at GitHub (https://github.com/chrisnic12/covid_cyprus).

## References

[1] Coronaviridae Study Group of the International Committee on Taxonomy of Viruses. The species severe acute respiratory syndrome-related coronavirus: classifying 2019-ncov and naming it sars-cov-2. *Nat. Microbiol.* **5**, 536–544 (2020). 10.1038/s41564-020-0695-z PMC7095448 32123347

[2] Zhu N (2020). A novel coronavirus from patients with pneumonia in China, 2019. N. Engl. J. Med..

[3] World Health Organization. *Covid-19 weekly epidemiological update* (Technical Report, 2021).

[4] Beigel JH (2020). Remdesivir for the treatment of Covid-19: preliminary report. N. Engl. J. Med..

[5] Polack FP (2020). Safety and efficacy of the bnt162b2 mrna covid-19 vaccine. N. Engl. J. Med..

[6] Baden LR (2020). Efficacy and safety of the mrna-1273 sars-cov-2 vaccine. N. Engl. J. Med..

[7] Voysey M (2021). Safety and efficacy of the chadox1 ncov-19 vaccine (azd1222) against sars-cov-2: an interim analysis of four randomised controlled trials in brazil, south africa, and the uk. Lancet.

[8] Kermack W, McKendrick AG (1927). A contribution to the mathematical theory of epidemics. Proc. R. Soc. London Ser. A.

[9] Schröder AL, Fryzlewicz P (2013). Adaptive trend estimation in financial time series via multiscale change-point-induced basis recovery. Stat. Its Interface.

[10] Bolton R, Hand D (2002). Statistical fraud detection: a review. Stat. Sci..

[11] Olshen AB, Venkatraman ES, Lucito R, Wigler M (2004). Circular binary segmentation for the analysis of array-based DNA copy number data. Biostatistics.

[12] Jandhyala V, Fotopoulos S, MacNeill I, Liu P (2013). Inference for single and multiple change-points in time series. J. Time Ser. Anal..

[13] Anastasiou, A. & Fryzlewicz, P. Detecting multiple generalized change-points by isolating single ones. https://arxiv.org/pdf/1901.10852.pdf (2019). 10.1007/s00184-021-00821-6 PMC8142888 34054146

[14] Kedem B, Fokianos K (2002). Regression Models for Time Series Analysis.

[15] Fokianos K, Davis R, Holan S, Lund R, Ravishanker N (2015). Statistical Analysis of Count Time Series Models: A GLM perspective. Handbook of Discrete-Valued Time Series, Handbooks of Modern Statistical Methods.

[16] McCullagh P, Nelder J. A (1989). Generalized Linear Models.

[17] Fokianos K, Tjøstheim D (2011). Log-linear poisson autoregression. J. Multivar. Anal..

[18] Liboschik T, Fokianos K, Fried R (2017). tscount: an R package for analysis of count time series following generalized linear models. J. Stat. Softw..

[19] Keeling MJ, Rohani P (2008). Modeling Infectious Diseases in Humans and Animals.

[20] Nicolaides C, Avraam D, Cueto-Felgueroso L, González MC, Juanes R (2020). Hand-hygiene mitigation strategies against global disease spreading through the air transportation network. Risk Anal..

[21] Peng, L., Yang, W., Zhang, D., Zhuge, C. & L., H. Epidemic analysis of COVID-19 in China by dynamical modeling. (2020). arXiv:2002.06563.

[22] Li R (2020). Substantial undocumented infection facilitates the rapid dissemination of novel coronavirus (sars-cov-2). Science.

[23] Bajardi P (2011). Human mobility networks, travel restrictions, and the global spread of 2009 h1n1 pandemic. PloS ONE.

[24] Nicolaides C, Cueto-Felgueroso L, González MC, Juanes R (2012). A metric of influential spreading during contagion dynamics through the air transportation network. PloS ONE.

[25] Bettencourt LM, Ribeiro RM (2008). Real time Bayesian estimation of the epidemic potential of emerging infectious diseases. PLoS ONE.

[26] Systrom, K. The metric we need to manage COVID-19 rt: The effective reproduction number. *Internet]*. http://systrom.com/blog/the-metric-we-need-to-managecovid-19 (2020).

[27] Bernardo JM, Smith AFM (1994). Bayesian Theory.

[28] Cori A, Ferguson NM, Fraser C, Cauchemez S (2013). A new framework and software to estimate time-varying reproduction numbers during epidemics. Am. J. Epidemiol..

[29] Ferguson, N., Laydon, M., Nedjati Gilani, N. *et al*. Impact of non-pharmaceutical interventions (NPI)s to reduce COVID–19 mortality and healthcare demand. 10.25561/77482 (2020). Imperial College London (16-03-2020).

