# Supplementary material for: Modeling the first wave of Covid-19 pandemic in the Republic of Cyprus
Source: Sci Rep. 2021 Apr 1;11:7342. doi: 10.1038/s41598-021-86606-3 (PMC8017012; doi:10.1038/s41598-021-86606-3)
Supplement: Supplementary file 1 — Supplementary Information. [file 41598_2021_86606_MOESM1_ESM.pdf]

Appendix:
Modeling the First Wave of Covid-19 Pandemic in the Republic of Cyprus

Sergios Agapiou[1,+], Andreas Anastasiou[1,+], Anastassia Baxevani[1,+], Christos Nicolaides[2,5,+], Georgios Hadjigeorgiou[3], Tasos Christofides[1], Elisavet Constantinou[4], Georgios Nikolopoulos[3,*], and Konstantinos Fokianos[1,*]

[1]Department of Mathematics & Statistics, University of Cyprus
[2]Department of Business & Public Administration, University of Cyprus
[3]Medical School, University of Cyprus
[4]Cyprus Ministry of Health
[5]Nireas Research Centre, University of Cyprus
[*]To Whom Correspondence: nikolopoulos.georgios@ucy.ac.cy & fokianos@ucy.ac.cy
[+]authors are listed in alphabetical order

February 26, 2021

A1 Isolate-Detect Methodology

The existing change-point detection techniques for the scenarios mentioned in the Change-point Analysis and Projections subsection of the main text are mainly split into two categories based on whether the change-points are detected all at once or one at a time. The former category mainly includes optimization-based methods, in which the estimated signal is chosen based on its least squares or log-likelihood criterion penalized by a complexity rule in order to avoid overfitting. The most common example of a penalty function is the Bayesian Information Criterion (BIC); see (8) and (9) for details. In the latter category, in which change-points are detected one at a time, a popular method is binary segmentation, which performs an iterative binary splitting of the data on intervals determined by the previously obtained splits. Even though binary segmentation is conceptually simple, it has the disadvantage that at each step of the algorithm, it looks for a single change-point in possibly long intervals, which leads to its suboptimality in terms of accuracy, especially for signals with frequent change-points. The Isolate-Detect (ID) methodology of (1), which is used for the analysis carried out in our paper, works towards solving this issue.

The concept behind ID is simple and is split into two stages; firstly, the isolation of each of the true change-points within subintervals of the domain $[1, 2, \ldots, T]$, and secondly their detection. The basic idea is that for an observed data sequence of length T and with λ_T a positive constant, ID first creates two ordered sets of $Q = \lceil T/\lambda_T \rceil$ right- and left-expanding intervals as follows. The jth right-expanding interval is $R_j = [1, j\lambda_T]$, while the jth left-expanding interval is $L_j = [T - j\lambda_T + 1, T]$. These intervals are collected in the ordered set $S_{RL} = \{R_1, L_1, R_2, L_2, \ldots, R_Q, L_Q\}$. For a suitably chosen contrast function, ID identifies the point with the maximum contrast value in R_1. If its value exceeds a threshold, denoted by ζ_T, then it is taken as a change-point. If not, then the process tests the next interval in S_{RL}. Upon detection, the algorithm makes a new start from the end-point (or start-point) of the right- (or left-) expanding interval where the detection occurred.

For clarity of exposition, we give below a simple example. Figure A1 covers a specific case of two change-points, $r_1 = 38$ and $r_2 = 77$. We will be referring to Phases 1 and 2 involving six and four intervals, respectively. These are clearly indicated in the figure and they are only related to this specific example, as for cases with more change-points will entertain more such phases. At the beginning, $s = 1$, $e = T = 100$, and we take the expansion parameter $\lambda_T = 10$. Then, r_2 gets detected in $\{X_{s^*}, X_{s^*+1}, \ldots, X_e\}$, where $s^* = 71$. After the detection, e is updated as the start-point of the interval where the detection occurred; therefore, $e = 71$. In Phase 2 indicated in the plot, ID is applied in $[s, e] = [1, 71]$. Intervals 1, 3 and 5 of Phase 1 will not be re-examined in Phase 2 and r_1 gets detected in $\{X_s, X_{s+1}, \ldots, X_{e^*}\}$, where $e^* = 40$. After the detection, s is updated as the end-point of the interval where the detection occurred; therefore, $s = 40$. Our method is then applied in $[s, e] = [40, 71]$; there will be no interval $[s^*, e^*] \subseteq [40, 71]$ on which the contrast function value exceeds ζ_T and therefore, the process will terminate.

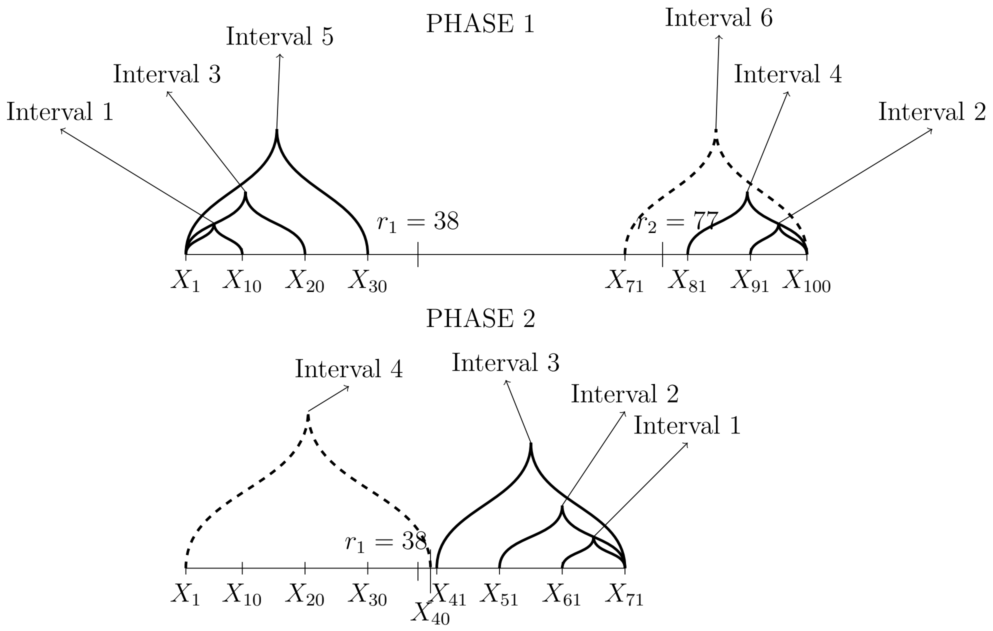

Figure A1: An example with two change-points; $r_1 = 38$ and $r_2 = 77$. The dashed line is the interval in which the detection took place in each phase.

A2 More on Count Time Series Models and Interventions

Recall model (2) of the main text and that the parameters d, a_1, b_1 can be positive or negative but they need to satisfy certain conditions so that we obtain stable behavior of the process. Note that the lagged observations of the response X_t are fed into the autoregressive equation for ν_t via the term $\log(X_{t-1} + 1)$. This is a one-to-one transformation of X_{t-1} which avoids zero data values. Moreover, both λ_t and X_t are transformed into the same scale. Covariates can be easily accommodated by model (2) of the main text. When $a_1 = 0$, we obtain an AR(1) type model in terms of $\log(X_{t-1} + 1)$. In addition, the log-intensity process of (2) in the can be rewritten as

$$\nu_t = d\frac{1 - a_1^t}{1 - a_1} + a_1^t \nu_0 + b_1 \sum_{i=0}^{t-1} a_1^i \log(1 + X_{t-i-1}),$$

after repeated substitution. Hence, we obtain again that the hidden process $\{\nu_t\}$ is determined by past functions of lagged responses, i.e. (2) belongs to the class of observation driven models; see (4).

Models like (2) of the main text, can accommodate various type of interventions (or extraordinary) observations by suitable modification. Generally speaking, intervention effects on time series data are classified according to whether their impact is concentrated on a single or a few data points, or whether they affect the whole process from some specific time $t = \tau$ on. In classical linear time series methodology an intervention effect is included in the observation equation by employing a sequence of deterministic covariates $\{W_t\}$ of the form

$$W_t = \xi(\mathcal{B}) I_t(\tau), \tag{AA1}$$

where $\xi(\mathcal{B})$ is a polynomial operator, $\mathcal{B}$ is the shift operator such that $\mathcal{B}^i W_t = W_{t-i}$ and $I_t(\tau)$ is an indicator function, with $I_t(\tau) = 1$ if $t = \tau$, and $I_t(\tau) = 0$ if $t \neq \tau$. The choice of the operator $\xi(\mathcal{B})$ determines the kind of intervention effect: additive outlier (AO), transient shift (TS), level shift (LS) and innovational outlier (IO). Since models of the form (2) in the main text are not defined in terms of innovations, we focus on the first three types of interventions. A model like (2), is determined by a latent process. Therefore a formal linear structure, as in the case of Gaussian linear time series model does not hold any more and interpretation of the interventions is a more complicated issue. Hence, a method which allows detection of interventions and estimation of their size is needed so that structural changes can be identified successfully. Important steps to achieve this goal are the following; see (2):

1. A suitable model for accommodating interventions in count time series data.

2. Derivation of test procedures for their successful detection.

3. Implementation of joint maximum likelihood estimation of model parameters and outlier sizes.

4. Correction of the observed series for the detected interventions.

All these issues and possible directions for further developments of the methodology have been addressed by (6) under the Poisson and mixed Poisson distributional framework.

A3 Computational Details for Fitting Equations (6) of the main text

According to the official reports, the number of quarantined cases (Q), recovered (R) and deaths (D), due to COVID-19, are available. However, the recovered and death cases are directly related to the number of quarantine cases, which plays an important role in the analysis, especially since the numbers of exposed (E) and infectious (I) cases are very hard to determine. The latter two are therefore treated as hidden variables. This implies that we need to estimate the four parameters $\zeta, \beta, \gamma^{-1}, \delta^{-1}$ and both the time dependent cure rate $\lambda(t)$ and mortality rate $\kappa(t)$. This is an optimization problem that we solve as follows: first we allow the latent time γ^{-1} to vary between 1 and 7 days and for each fixed γ^{-1}, we explore its influence on the rest of the parameters. The system of differential equations (6) in the main text is solved numerically using the Runge-Kutta 45 numerical scheme. The left plot of Figure A2 shows that the protection rate ζ and the transmission rate β both attain their corresponding maximum value when γ^{-1} is equal to 3 days. Note that ζ takes values between 0.08 and 0.2, while β converges very fast to 1. The reciprocal of the quarantine time δ^{-1} is increasing with the latent time γ^{-1}. One would suspect that longer latent time results to higher transmission rate and as the latent time increases almost every unprotected person will be infected after a direct contact with a COVID-19 patient. The right plot of Figure A2 shows the effect of the latent time on the total number of infected cases (exposed and infectious $E(t) + I(t)$) but not yet quarantined. The peak of the infection was achieved between the 21st and the 24th of March, depending on the latent time with the estimated number of infected people ranging between 338 and 526, depending again on the latent time considered. Hence, once the latent time γ^{-1} is fixed, the fitting performance depends on the values

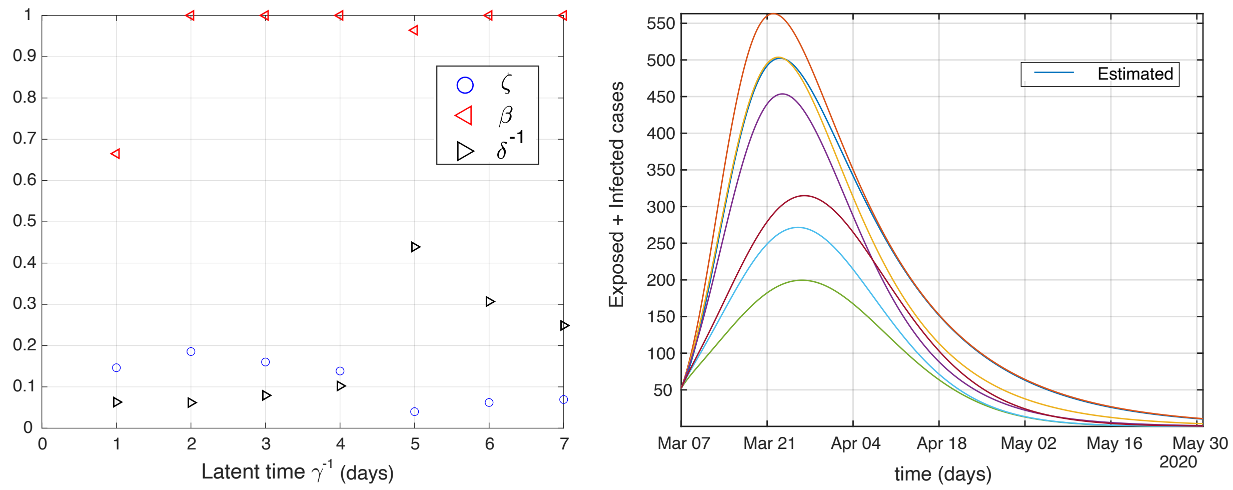

Figure A2: Sensitivity analysis on the parameters for the model defined by (6) in the main text. The influence of the latent time γ^{-1} on the protection rate ζ, the transmission rate β and the quarantine time δ^{-1} (left plot), the sum of exposed and infectious cases $E(t) + I(t)$ (right plot).

of ζ, β and δ^{-1}. After a small sensitivity analysis the latent time was finally determined as 3 days. The mortality rate $\kappa(t)$ is constantly very small and almost equal to zero, therefore we have not attempted to fit any function to it. For the cure rate $\lambda(t)$ we have fitted the exponential function given in (9) in the main text, the idea behind being that with time the recovery should converge to a constant rate. For the parameter estimation we have used a modified version of the MATLAB code given by (3) because Cyprus is a small country and this fact needs to be taken properly into account.

A4 Prior Modelling and Computational Details for the Estimation of the effective Reproduction Number R_t section of the main text

We present a Bayesian analysis, for the model defined by (4) in the Compartmental Model 1 subsection of the main text, for the parameters α and β, based on six separate fortnight periods. We use Beta prior distributions on the reporting rate $\alpha \in [0, 1]$, with parameters reflecting the amount of targeted and random testing performed in Cyprus, at the time under consideration. In particular, in the first period (when the number of tests was relatively low) we employ a symmetric prior around the value $\alpha = 0.5$, while for later periods (when the number of targeted and random tests increased) we let the prior become progressively skewed towards 1. For the transmission rate $\beta > 0$, in the first period we use a $Gamma(3/2, 3/2)$ prior, which puts high probability around 2, while for later periods we use an $Exponential(1)$ prior which puts more mass closer to zero. This choice reflects the existence of super-spreaders in the early stages of the outbreak with higher probability compared to later on.

In each time-period under consideration we also need to initialize the outbreak in Cyprus. For the first period in both datasets, we use a uniform prior supported in $\{0, 1, \ldots, 10\}$ on the number of exposed and

the number of undocumented infected 3 days before the first recorded incident. The two priors are independent, while the number of susceptible individuals is taken equal to Cyprus' population and the number of infected-reported equal to zero. For later periods, we use as priors on the four state variables, their posterior distributions at the end of the previous period (corrected appropriately based on the observation at the end of the previous period).

Following (5), we assume that the daily number of reported cases are independent Gaussian random variables and use an empirical variance given as

$$\sigma^2(t) = \max\Big(1, \frac{y(t)^2}{4}\Big),$$

by recalling that $y(t)$ denotes the number of infected cases at day t. This allows us to build a Gaussian likelihood for the parameters α and β. Combining this likelihood with the prior distributions, we can deduce a formula for the posterior distribution on α, β. This distribution is not available in a closed form, hence in order to compute posterior estimates and their respective uncertainty quantification, we need to sample it. In the relatively simple setting of Model 1, it is feasible to employ Markov chain Monte Carlo methods, (see (7)), in order to sample the posterior (namely, we use an independence sampler). This is in contrast to the model defined by (5) in the Compartmental Model 2 subsection of the main text, see (5), where one has to use the Ensemble Adjustment Kalman Filter (EAKF), which introduces some approximations to the posterior distribution, due to the more complex meta-population structure. Originally developed for use in weather prediction, the EAKF assumes a Gaussian distribution for both the prior and the likelihood and adjusts the prior distribution to a posterior using Bayes rule deterministically. In particular, the EAKF assumes that both the prior distribution and likelihood are Gaussian, and thus can be fully characterized by their first two moments (mean and variance). The update scheme for ensemble members is computed using Bayes rule (posterior $\sim$ prior $\times$ likelihood) via the convolution of the two Gaussian distributions (see (5) for the implementation).

A5 Results of Change-Point Analysis for Piecewise-Constant Model

We report the results obtained after fitting a piecewise-constant signal plus noise model, as described in the Change-point Analysis and Projections subsection of the main text. The scenario here is that at each change-point, we have a sudden jump in the mean level of the signal. Figure A3 below gives a graphical representation of the relevant change-point analysis carried out for the daily number of COVID-19 cases. Based on this piecewise-constant scenario, the Isolate-Detect methodology has detected five important changes leading to six homogeneous periods in terms of the average number of detected cases per day. Changes are detected on the 11th and the 25th of March, on the 2nd and the 14th of April, as well as on the 1st of May. The first change-point on the 11th of March introduces a jump of magnitude 10.56 (accuracy is up to two decimal places), meaning that in the second period there is a mean growth of about 11 detected cases compared to the first period. The initial period mainly consists of days without any new cases detected and therefore, the first change-point is related to the outbreak of the epidemic in the society. The second change-point on the 25th of March indicates an elevation of the number of cases by 23.21, which is very

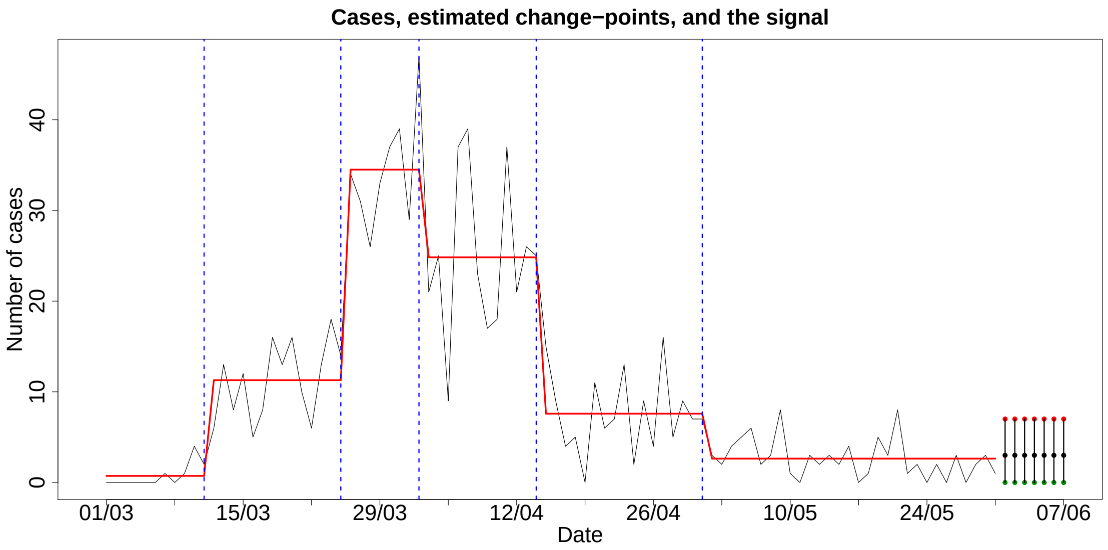

Figure A3: The real data (black coloured line) and the estimated piecewise-constant fit (red coloured line) for the daily incidence rate. The change-point locations are given with dotted, blue vertical lines. At the right-end of the plot point estimators (black dots) and 95% prediction intervals for the number of daily cases for the next week are reported.

important and captures the development of the clusters A and B as mentioned in the Descriptive surveillance statistics subsection of the main text. The third and fourth change-points, detected on the 2nd and 14th of April show a reduction in the number of cases, with the number falling by 9.67 and 17.24, respectively. Both these change-points show the importance (in fighting the virus) of the Government's decrees on the 24th and 31st of March for a general lockdown. The last change-point indicates a further reduction in the number of cases with magnitude 4.95. Corresponding predictions obtained by this model show that there will be no more than 7 new cases per day with the point estimator being equal to 3 cases per day. Our analysis under piecewise-constancy gives two more change-points than those detected when the piecewise-linear structure was employed in the Change-point Analysis subsection of the main text; in fact, it is always expected to detect more change-points under piecewise-constancy than under piecewise-linearity. For example, think of a noiseless linear signal with upward trend that does not have any change-points in the first derivative. Treating this signal under the piecewise-constant scenario, would mean that each data point is a change-point because a jump of magnitude equal to the slope of the signal is introduced at every time point.

A6 Additional Figures

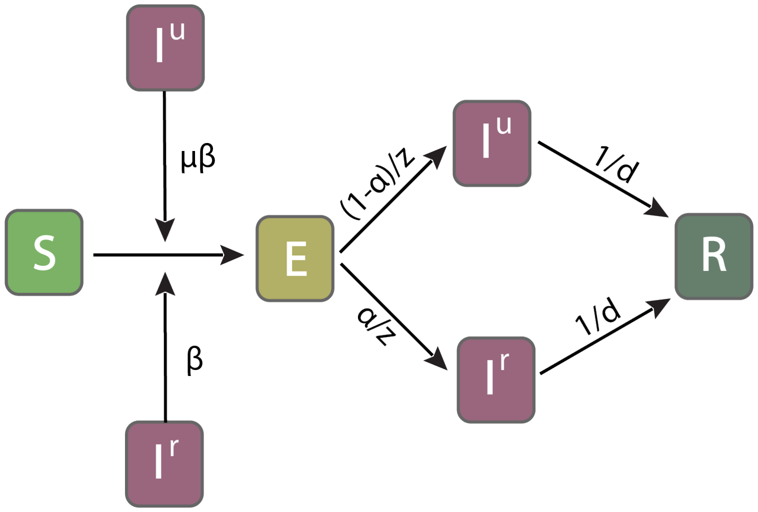

Figure A4: Representation of the SEIR model as described by (5).

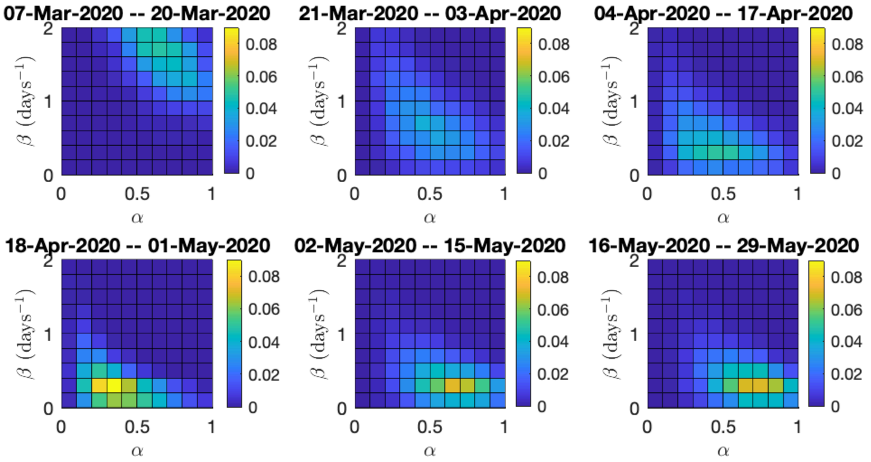

Figure A5: Joint posterior distributions of the reporting rate α and the transmission rate β in Model 1, for the six fortnight periods starting from 07/03/2020 until 29/05/2020. Analysis using data on local transmission only.

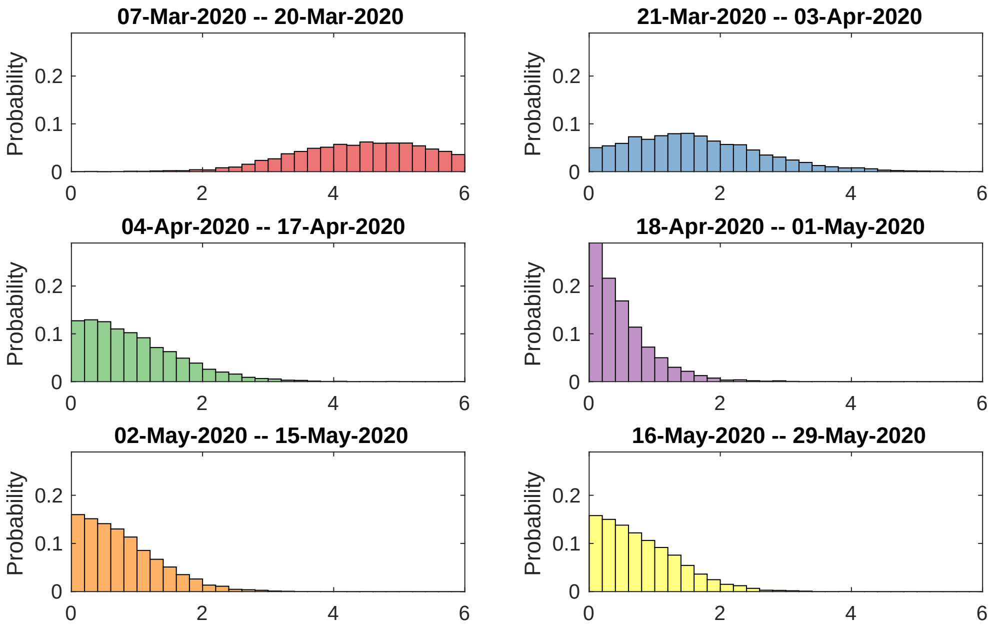

Figure A6: Posterior distributions of the effective reproductive number in Model 1, for the six fortnight periods starting from 07/03/2020 until 29/05/2020. Analysis using data on local transmission only.

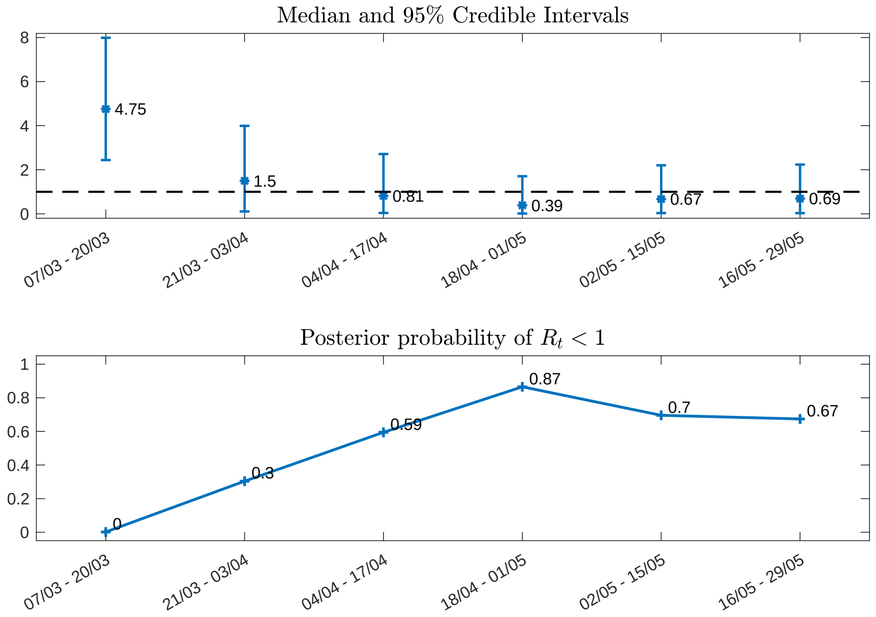

Figure A7: Median and 95% credible intervals for the posterior distributions of the effective reproduction number in Model 1, for the six fortnight periods starting from 07/03/2020 until 29/05/2020 (top). Posterior probabilities of the event $R_t < 1$ (bottom). Analysis using data on local transmission only.

Data availability

Data and code are available at GitHub (https://github.com/chrisnic12/covid_cyprus)

References

[1] Anastasiou, A. and P. Fryzlewicz (2019). Detecting multiple generalized change-points by isolating single ones. https://arxiv.org/pdf/1901.10852.pdf.

[2] Chen, C. and L.-M. Liu (1993). Joint estimation of model parameters and outlier effects in time series. Journal of the American Statistical Association 88, 284–297.

[3] Cheynet, E. (2020). Echeynet/SEIR: Generalized SEIR epidemic model (fitting and computation). url = https://zenodo.org/record/3819519.

[4] Cox, D. R. (1981). Statistical analysis of time series: Some recent developments. Scandinavian Journal of Statistics 8, 93–115.

[5] Li, R., S. Pei, B. Chen, Y. Song, T. Zhang, W. Yang, and J. Shaman (2020). Substantial undocumented infection facilitates the rapid dissemination of novel coronavirus (sars-cov-2). Science 368(6490), 489–493.

[6] Liboschik, T., K. Fokianos, and R. Fried (2017). tscount: An R package for analysis of count time series following generalized linear models. Journal of Statistical Software 82, 1–51. DOI: https://www.jstatsoft.org/article/view/v082i0510.18637/jss.v082.i05.

[7] Robert, C. and G. Casella (2013). Monte Carlo statistical methods. Springer Science & Business Media.

[8] Schwarz, G. (1978). Estimating the dimension of a model. Annals of Statistics 6, 461–464.

[9] Yao, Y.-C. (1988). Estimating the number of change-points via Schwarz' criterion. Statistics & Probability Letters **6**, 181–189.
